# Diaphragm Based Fiber Bragg Grating Acceleration Sensor with Temperature Compensation

**DOI:** 10.3390/s17010218

**Published:** 2017-01-23

**Authors:** Tianliang Li, Yuegang Tan, Xue Han, Kai Zheng, Zude Zhou

**Affiliations:** School of Mechanical and Electronic Engineering, Wuhan University of Technology, Wuhan 430070, China; ygtan@whut.edu.cn (Y.T.); hanxue@whut.edu.cn (X.H.); zhengkai2001@163.com (K.Z.); zudezhou@whut.edu.cn (Z.Z.)

**Keywords:** fiber Bragg grating (FBG), temperature compensation, vibration sensor, diaphragm

## Abstract

A novel fiber Bragg grating (FBG) sensing-based acceleration sensor has been proposed to simultaneously decouple and measure temperature and acceleration in real-time. This design applied a diaphragm structure and utilized the axial property of a tightly suspended optical fiber, enabling improvement in its sensitivity and resonant frequency and achieve a low cross-sensitivity. The theoretical vibrational model of the sensor has been built, and its design parameters and sensing properties have been analyzed through the numerical analysis. A decoupling method has been presented with consideration of the thermal expansion of the sensor structure to realize temperature compensation. Experimental results show that the temperature sensitivity is 8.66 pm/°C within the range of 30–90 °C. The acceleration sensitivity is 20.189 pm/g with a linearity of 0.764% within the range of 5~65 m/s^2^. The corresponding working bandwidth is 10~200 Hz and its resonant frequency is 600 Hz. This sensor possesses an excellent impact resistance for the cross direction, and the cross-axis sensitivity is below 3.31%. This implementation can avoid the FBG-pasting procedure and overcome its associated shortcomings. The performance of the proposed acceleration sensor can be easily adjusted by modifying their corresponding physical parameters to satisfy requirements from different vibration measurements.

## 1. Introduction

Compared with the traditional electronic vibration detection sensors, Fiber Bragg grating (FBG)-based vibration sensors provide an emerging and practical alternative solution [1,2,3]. They have offered the advantages of miniature size and light weight, are resistant to electromagnetic interference and corrosion, and easy to perform distributed dynamic measurements [1,2,3,4,5]. In recent years, FBG-derived sensors have aroused increased interest for structural health monitoring in many industrial sectors due to their remarkable attributes [6,7]. FBGs have been increasingly extended to measure seismic and mechanical vibration through designing mechanical structures to convert vibration into strain signals [8]. Au et al. presented a tapered plate FBG accelerometer with a sensitivity of 18.93 με/g (Note: g represents the gravitational acceleration) [9]. However, this sensor suffered from a low resonant frequency of 150 Hz. Some scholars have proposed some FBG accelerometers based on using cantilever beam structures or other improved beam forms to improve their sensing properties [10,11]. Basumallick et al. presented to improve the sensitivity of a FBG-based vibration sensor by changing the distance between the FBG element axis and the neutral axis of the cantilever beam [10]. However, the resonant frequency of this design was still limited to 150 Hz. In order to improve the resonant frequency of the pasted FBG acceleration sensors, Liu et al. presented a novel diaphragm-based FBG accelerometer with the FBG element pasted around the circumferential direction on the diaphragm [12]. This design provided a linear response over a frequency ranging from 10 to 200 Hz with a sensitivity of 36.6 pm/g. All of the above-mentioned FBG-based vibration sensors involved pasting FBG elements on the surface of mechanical structure elastomers. However, there are several drawbacks associated with these FBG-pasting vibration sensors, such as poor repeatability, chirp failure, and low resonant frequency.

To overcome these limitations, the use of the axial property of a suspended or metal-coated optical fiber has been proposed [13,14]. Antunes et al. proposed an FBG-based accelerometer with a low cross-sensitivity based on the use of axial property, and two optical fibers were arranged in a parallel manner on an improved cantilever beam [14]. The resonant frequency of this design was improved up to 344 Hz. Although the drawbacks of pasted FBG vibration sensors can be overcome by using the axial property of optical fiber to design vibration sensors, these implementations still suffered from poor dynamic properties, such as low resonant frequency and limited bandwidth [13,14,15]. In our previous work, we have proposed a new method that was based on the use of the transverse property of a suspended optical fiber to design an FBG vibration sensor [16]. This design can also avoid the FBG-pasting process and its accompanied limitations, and provided a higher sensitivity. However, its resonant frequency is 34 Hz, making it difficult to meet the requirements of complex mechanical vibration situations.

To address these issues, this paper has proposed an FBG sensing-based acceleration sensor to simultaneously measure temperature and medium-high frequency vibration with a high resonant frequency, and support multi-point distributed sensing. The axial property of a tightly suspended optical fiber has been utilized as elastomer together with the suspended diaphragm to sense the acceleration of target. The optical fiber inscribed with FBG elements was compressed and stretched to sense the strain caused by vibration. Such combined configuration can effectively overcome the limitations accompanied with the pasting process and improve the sensitivity and resonant frequency, leading to achieving a low cross-sensitivity and avoiding the chirping failure. The designed sensor consists of a diaphragm and an optical fiber that was fixed between the diaphragm center and the sensor housing base. Two FBGs were inscribed in the same optical fiber to decouple vibration and temperature. The working principle and theoretical modeling of the proposed sensor have been introduced and derived, and the corresponding numerical and experimental analyses have been performed to improve and validate the proposed design. This paper is organized as the following sections: working principle and modeling of the proposed sensor, numerical analysis and structure design, experiments and discussion, and conclusion.

## 2. Working Principle and Modeling of the Proposed Sensor

**Figure 1:**
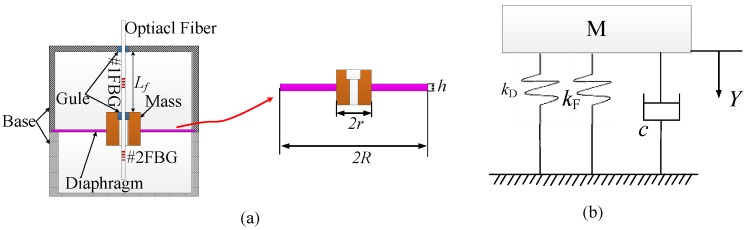
Schematic and modeling of the proposed sensor: (**a**) the schemetic design of the FBG-based vibration sensor; and (**b**) the vibrational modeling of this proposed sensor.

The schemetic design of the proposed sensor and its simplified model are shown in Figure 1. This sensor mainly consists of a mass, a diaphragm, and a suspended optical fiber embedded with two FBGs, as shown in Figure 1a. The mass was assembled in the center of the suspended diaphram, and the optical fiber was suspended with two ends fixed on the sensor housing frame and the upper suface of the mass. While measuring the vibration, the inertial force of the mass can compress or stretch #1FBG, which was embedded in the optical fiber and arranged between the sensor frame and the mass. Thus, vibration can be detected using the relative center wavelength shift of #1FBG. The other FBG element of #2FBG in the optical fiber was suspended in interior of the sensor frame, and it was used to measure and decouple the temperature effect.

### 2.1. Temperature Sensing Model

According to the working principle of FBG sensing, the relationship between the center wavelength shift of #2FBG and temperature can be written as:

(1) $$\frac{\Delta \lambda_{2}}{\lambda_{2}}=\left(\alpha_{f}+\xi_{f}\right)\Delta T$$

where Δ*λ*_2_ is the center wavelength shift of #2FBG; *λ*_2_ represents the center wavelength of #2FBG. ζ*_f_* denotes the thermo-optic coefficient of the used optical fiber. α*_f_* expresses the thermal expansion coefficient of FBG, and Δ*T* is the temperature variation.

The suspended optical fiber posseses a small diameter, and will be compressed or stretched along the axial direction with temperature variation. Therefore, the thermal expansion effect of this sensor can be simplified as a linear model along the axial direction of the optical fiber. The equivalent linear thermal expansion coefficient of the sensor base and the diaphragm along the axial direction of optical fiber can be denoted as *α_M_*. Consequently, the relationship among the center wavelength shift of #1FBG, temperature and strain can be expressed as [17]:

(2) $$\frac{\Delta \lambda_{1}}{\lambda_{1}}=\left(1-\rho_{e}\right)\Delta \varepsilon +\left(\alpha_{f}+\alpha_{M}+\xi_{f}\right)\Delta Τ$$

where Δ*λ*_1_ is the center wavelength shift of #1FBG; *λ*_1_ represents the center wavelength of #1FBG. *ρ_e_* denotes the strain-optic coefficient of the optical fiber, and Δ*ε* is the axial strain increment of #1FBG under the inertial force. *α_M_* expresses the equivalent linear thermal expansion coefficient of the sensor base and the diaphragm along the *y* direction.

Combing Equations (1) and (2), the temperature variation Δ*T* can be obtained through Δ*λ*_2_, but the strain Δ*ε* cannot be decoupled by using these two equations. Assuming that there is no vibration stimulation, the center wavelength shifts Δ*λ*_10_ of #1FBG will only be affected by temperature. Therefore, a new relationship between the Δ*λ*_1_ and strain Δ*ε* can be derived as:

(3) $$\Delta \lambda_{1}=\left(1-\rho_{e}\right)\Delta \varepsilon \lambda_{1}+\frac{\lambda_{1}\left(\alpha_{f}+\alpha_{M}+\xi_{f}\right)}{\lambda_{2}\left(\alpha_{f}+\xi_{f}\right)}\Delta \lambda_{2}$$

### 2.2. Vibration Sensing Model

The mechanical vibration model of this sensor can be simplified as a mass-spring-damper system with one degree of freedom (refer to Figure 1b). According to Newton’s second law, the kinetic equation of this sensor can be expressed as [18]:

(4) $$M\ddot{Y}+c\dot{Y}+\left(k_{D}+k_{F}\right)Y=Fsin(wt)$$

where, *Y* represents the deformation of the diaphragm, and *k_D_* is the transverse stiffness of the diaphragm. *c* denotes the damping ratio. *F* and *w* are the amplitude and frequency of the stimulation forces, respectively. *M* represents the mass weight, and *k_F_* is the axial stiffness of the optical fiber, which can be formulated as [19]:

(5) $$k_{F}=\frac{E_{f}A_{f}}{L_{f}}$$

where, *E_f_* and *A_f_* are Young’s modulus and the cross-section area of the optical fiber, respectively. *L_f_* represents the effective working length of the optical fiber.

According to elastic mechanics, the relationship between diaphragm deformation *Y* and the stimulation force *F* can be determined as [19,20]:

(6) $$Y=\frac{3\left(1-\mu^{2}\right)}{4\pi }\frac{FR^{2}}{Eh^{3}}\left[1-{\left(\frac{r}{R}\right)}^{2}\frac{1-{\left(\frac{r}{R}\right)}^{2}+4\ln^{2}(\frac{r}{R})}{1-{\left(\frac{r}{R}\right)}^{2}}\right]$$

where, *μ* and *E* represent Poisson ratio and Young’s Modulus of the diaphragm. *h*, *R*, and *r* denote the thickness, radius, and hard core radius of the diaphragm, respectively. According to Equation (6), the stiffness *k_D_* can be described as [19]:

(7) $$\frac{1}{k_{D}}=\frac{3\left(1-\mu^{2}\right)}{4\pi }\frac{R^{2}}{Eh^{3}}\left[1-{\left(\frac{r}{R}\right)}^{2}\frac{1-{\left(\frac{r}{R}\right)}^{2}+4\ln^{2}(\frac{r}{R})}{1-{\left(\frac{r}{R}\right)}^{2}}\right]$$

According to the definition of resonant frequency in mechanical vibrations, the resonant frequency *w_n_* of this sensor can be expressed as [18]:

(8) $$w_{n}=\sqrt{\frac{K}{M}}=\sqrt{\frac{k_{D}+k_{F}}{M}}$$

Consequently, the steady-state solution of Equation (4) can be determined as:

(9) $$Y=\frac{F/K}{\sqrt{4\xi^{2}\frac{w^{2}}{w_{n}}+{\left(1-\frac{w^{2}}{w_{n}}\right)}^{2}}}$$

The stimulation force *F* is equal to the inertial force *Ma*. When *w* << *w_n_*, Equation (9) can be simplified as:

(10) $$Y=\frac{a}{w_{n}}$$

where, *a* represents the measured acceleration along the *y* direction. The incremental length Δ*L* of #1FBG is equal to the diaphragm deformation *Y*, consequently the strain Δ*ε* of #1FBG can be expressed as:

(11) $$\Delta \varepsilon =\frac{\Delta L}{L_{f}}=\frac{Ma}{E_{f}A_{f}+K_{D}L_{f}}$$

Combining Equations (3) and (11), the center wavelength shift Δ*λ*_1_ of #1FBG can be reformulated as:

(12) $$\Delta \lambda_{1}=\frac{M\lambda_{1}\left(1-\rho_{e}\right)}{E_{f}A_{f}+K_{D}L_{f}}a+\frac{\lambda_{1}\left(\alpha_{f}+\alpha_{M}+\xi_{f}\right)}{\lambda_{2}\left(\alpha_{f}+\xi_{f}\right)}\Delta \lambda_{2}$$

Based on Equation (12), the temperature and acceleration can be decoupled and simultaneously detected by using two FBGs. Also according to Equation (12), the peak-valley sensitivity of this sensor for detecting acceleration *a* can be expressed as:

(13) $$S=\frac{M\lambda_{1}\left(1-\rho_{e}\right)}{E_{f}A_{f}+K_{D}L_{f}}$$

## 3. Numerical Analysis for Structure Design

**Figure 2:**
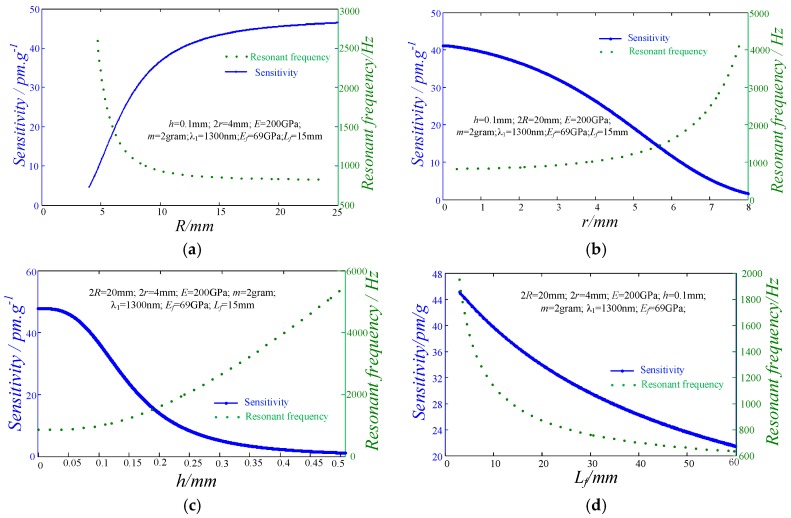
Relationship between the peak-valley sensitivity/resonant frequency and the configuration parameters of this sensor. (**a**) The effects of diaphragm radius on the sensor’s sensitivity and resonance frequency; (**b**) The effects of radius of the hard-core diaphragm on the sensor’s sensitivity and resonance frequency; (**c**) The effects of diaphragm thickness on the sensor’s sensitivity and resonance frequency; (**d**) The effects of the effective length of the optical fiber on the sensor’s sensitivity and resonance frequency.

The relationship between the sensitivity/resonant frequency and the configuration parameters of this sensor should be investigated to support the design improvement and structural optimization. According to Equations (8), (12) and (13), the relationship between the parameters, such as diaphragm radius, radius of the hard-core diaphragm, diaphragm thickness, and effective working length of the optical fiber, and the sensor’s sensitivity and resonant frequency has been determined, as shown in Figure 2. The results indicate that: (*i*) the sensitivity of this sensor increases with the increment of the diaphragm radius, but its resonant frequency decreases with the increase of the diaphragm radius. When the diaphragm radius is larger than 15 mm, both of the sensor’s sensitivity and resonant frequency are close to a constant, as illustrated in Figure 2a. Consequently, the diaphragm radius should be kept below 15 mm for performance adjustment; (*ii*) when the radius of the hard core diaphragm is less than 5 mm, the resonant frequency is almost unchanged, as demonstrated in Figure 2b. Therefore, the sensitivity of this sensor can be adjusted without sacrificing the resonant frequency when this radius value is less than 5 mm; (*iii*) when the diaphragm thickness resides in the range of less than 0.05, the sensing properties are difficult to be improved, as shown in Figure 2c; (*iv*) when the thickness is larger than 0.05 mm, the changing rate of the sensitivity and resonant frequency are greater than these in the other ranges; and (*v*) compared with the above parameters, the variation of the optical fiber length produces more significant influences on the sensor’s sensitivity and resonant frequency, as shown in Figure 2d.

**Figure 3:**
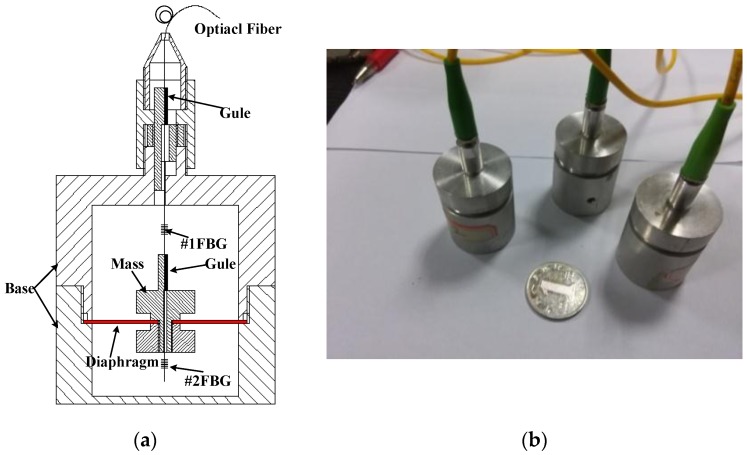
Assembly diagram and prototype of the proposed sensor: (**a**) 2D mechanical assembly drawing of the proposed sensor; and (**b**) physical prototypes of the proposed sensors.

Based on the above analysis, a stainless steel diaphragm was chosen, and it has an external diameter of 20 mm, a hard core diameter of 4mm and a thickness of 0.1 mm. The mass weight is 2 g, and the effective working length of optical fiber is determined as 15 mm. The initial center wavelengths of the #1FBG and #2FBG are 1297.989 nm and 1307.881 nm, respectively. In order to improve the compressive property of the optical fiber and enhance the measurement range of compression force, the optical fiber has been configured with the pre-stress, which caused the center wavelength of #1FBG to shift 506 pm. The schematic structure design and fabricated prototypes of the sensor are shown in Figure 3. Substituting these parameters into Equations (8) and (13), the resonant frequency and peak-valley sensitivity are, respectively, determined as 967.7 Hz and 36.56 pm/g.

## 4. Experiments and Discussion

### 4.1. Characterization of Temperature Effect

**Figure 4:**
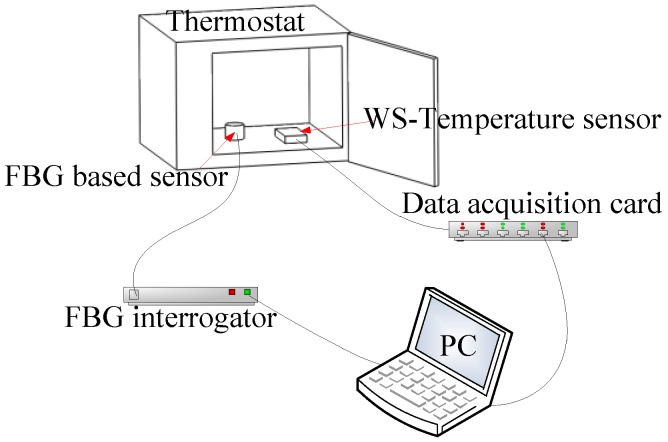
Schematic diagram for characterization of temperature effect.

In order to characterize the temperature effect of this sensor, the experimental setup has been built as shown in Figure 4. The commercial WS-type GWD50 temperature sensor was employed as a standard reference. The signals of the FBG-based vibration sensor and the commercial WS-type temperature sensor (range: 0~100 °C; fitted equation: voltage = 0.04001 × T + 1.00262) were collected using an FBG interrogator (high-speed FBG interrogator, Wuhan Wutos Co., Ltd., Wuhan, China) and a NI acquisition card (USB-6351, National Instrument, Austin, TX, USA), respectively. The experiments have been repeated three times with the temperature changing in the range of 30~90 °C.

**Figure 5:**
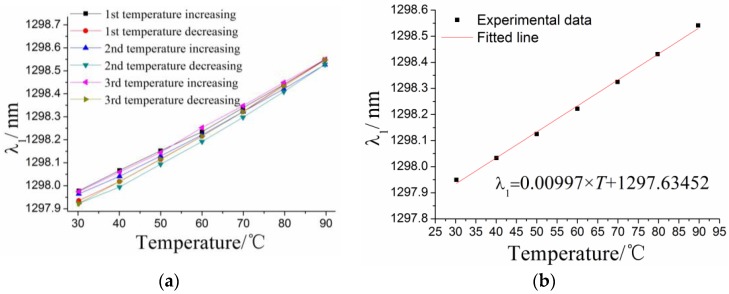
Relationship between the center wavelength of #1FBG *λ*_1_ and the temperature: (**a**) the experimental data for the the center wavelength of #1FBG *λ*_1_ and the temperature; and (**b**) the linearly fitted curve for the experimental data.

**Figure 6:**
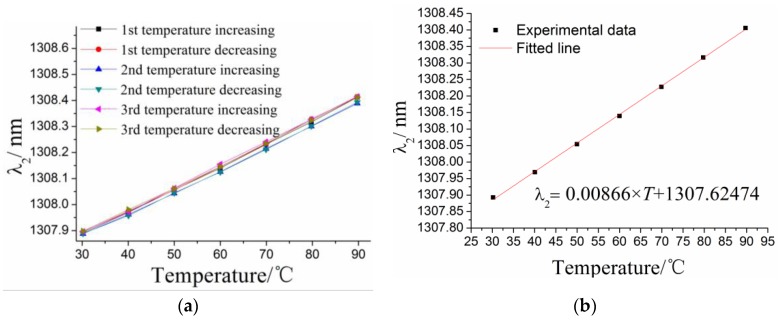
Relationship between the center wavelength *λ*_2_ of #2FBG and the temperature: (**a**) the experimental data for the the center wavelength of #2FBG *λ*_2_ and the temperature; and (**b**) the linearly fitted curve for the experimental data.

The relationship between the center wavelength *λ*_1_ of #1FBG and the environmental temperature has been determined as shown in Figure 5. The results suggest that there is a linear relationship between them. The relationship between the center wavelength *λ*_2_ of #2FBG and the temperature can be obtained using the same method, which is shown in Figure 6. Temperature effect characteristics of the two FBGs are shown in Table 1. The results suggest that the temperature sensitivity of the #1FBG is 9.97 pm/°C, greater than 8.66 pm/°C of #2FBG. This conclusion matches the theoretical analysis that the response sensitivity of #1FBG is greater than that of #2FBG according to Equations (1) and (3). It also indicates that the temperature influence coupling on #1FBG cannot be completely eliminated by two different FBGs.

### 4.2. Characterization of Static Properties

**Figure 7:**
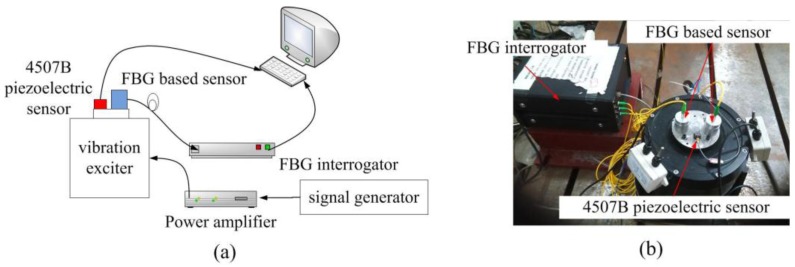
Schematic diagram and experimental setup for static experiments: (**a**) the schematic diagram for the static experiments; and (**b**) the experimental setup for static characterization.

The schematic diagram and hardware configuration for static experiments are shown in Figure 7. The exciting signals with different frequencies and amplitudes were generated by an AFG-2005 signal generator, and then were amplified by a power amplifier to drive the exciter to produce stimulations. The 4507-piezoelectric sensor with a sensitivity of 9.91 mv/m·s^−2^ was employed as a reference to calibrate the proposed FBG-based vibration sensor. Both of the piezoelectric sensor and the designed sensor were mounted on the vibration exciter.

**Figure 8:**
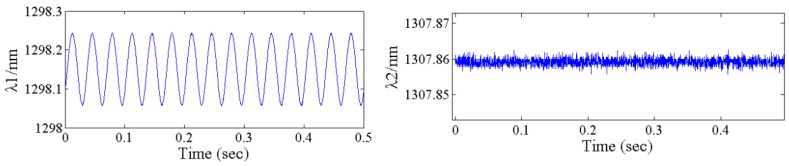
Reflective wavelength response of the #1FBG and #2FBG under a stimulation amplitude of 60 m/s^2^.

The amplitude of stimulation acceleration changed from 5 m/s^2^ to 65 m/s^2^, and the corresponding frequency was always kept at 30 Hz. The experiment has been repeated four times. The temperature of the whole process in the static experiments fluctuated around 27.4 °C, which can be regarded as a constant temperature. Combining the two fitted equations of temperature effects on the two FBGs, the center wavelength shift ∆*λ*_10_ of #1FBG can be determined as: ∆*λ*_10_ = 1.15 *× λ*_2_ *−* 1505.7026. Figure 8 shows the response of #1FBG and #2FBG under the stimulation amplitude of 60 m/s^2^. The response of #1FBG is a clearly sinusoid signal. However, there is almost no reflective wavelength response from #2FBG, because #2FBG was suspended and only affected by temperature variation.

**Figure 9:**
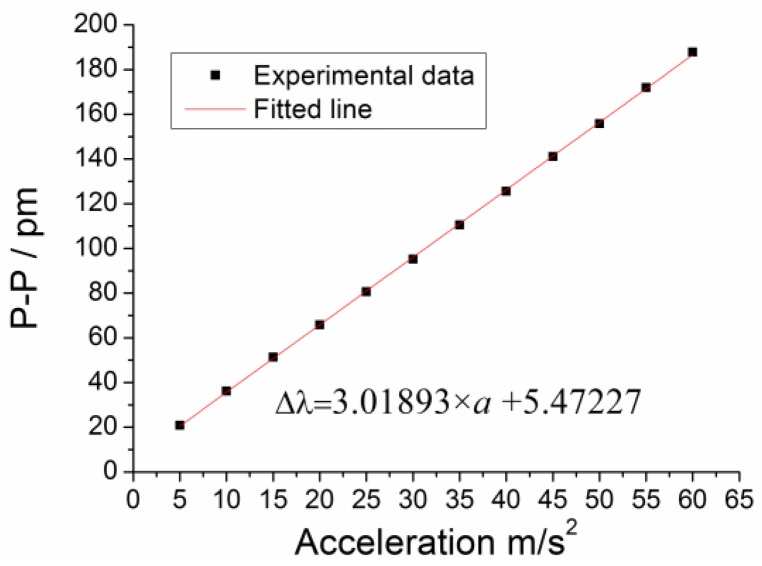
Relationship between the center wavelength shift ∆*λ*_1_ of #1FBG and acceleration.

In order to investigate the vibration characteristics of this sensor, the relationship between the average values of the experimental data and the applied acceleration *a* is determined as shown in Figure 9. The results show that: (i) the sensitivity of this sensor is 3.01 pm/m·s^−2^ with a linearity of 0.764%, which is basically consistent with the numerical analysis value of 3.656 pm/m·s^−2^; (ii) the fitted equation is ∆*λ*_1_ = 3.01893 *× a* + 5.47227.

Combining ∆*λ*_1_ with ∆*λ*_10_, a new equation for determination of the center wavelength shift of the FBGs and acceleration without temperature effects can be expressed as ∆*λ*_1_ − (1150 × *λ*_2_ *–* 1,505,702.6) = 3.01893 × *a* + 5.47227.

### 4.3. Characterization of Dynamic Properties

**Figure 10:**
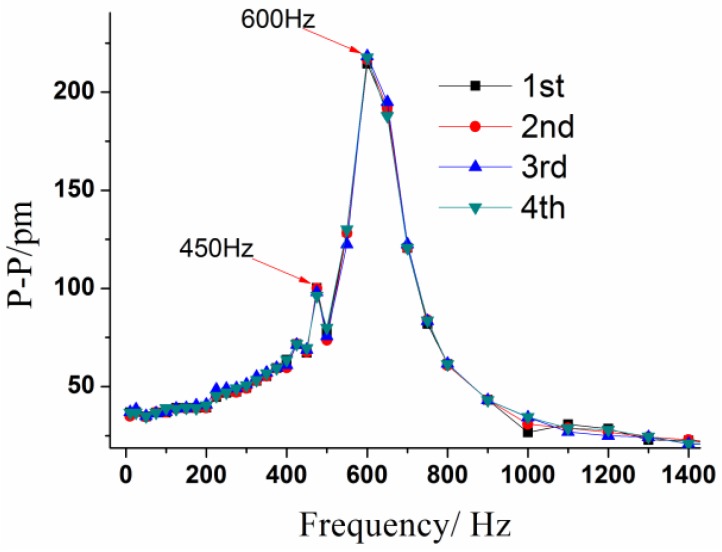
Frequency-amplitude response curves of this sensor.

The amplitude of vibration exciting acceleration was set at 10 m/s^2^ with the frequency increasing from 10 to 1500 Hz to investigate the dynamic properties of this sensor. The experiment was repeated four times. The corresponding frequency-amplitude response curves are shown in Figure 10. These results indicate that (i) when frequency is within the range of 10~200 Hz, the curve is almost parallel to horizontal axis, suggesting this sensor works in a linear working bandwidth; (ii) The peak-to-peak value reaches its maximum at approximately 600 Hz, which represents the resonant frequency of this sensor according to Equation (8).

**Figure 11:**
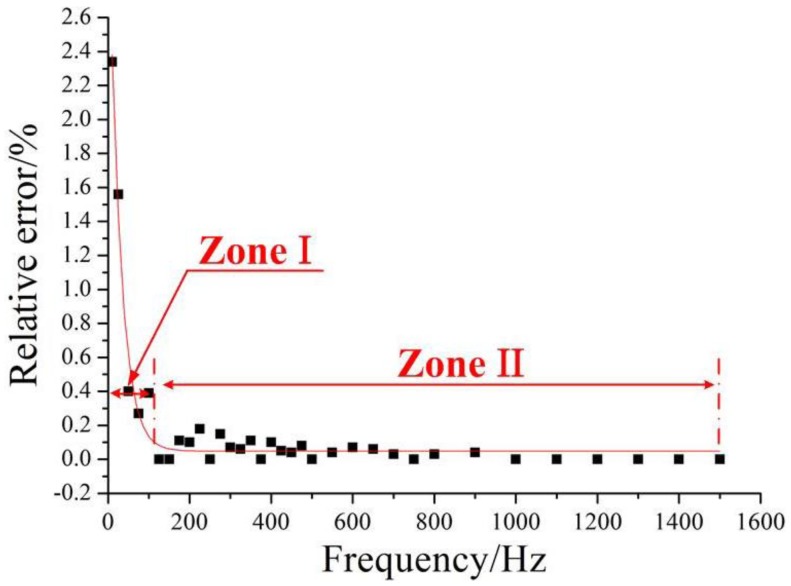
The measured frequency errors between the proposed sensor and the commercial piezoelectric sensor.

The errors of the measured frequencies between the proposed FBG vibration sensor and the commercial piezoelectric sensor have been displayed in Figure 11. The results indicate that when the frequency is within 10~100 Hz (zone-I), the maximum relative error is about 2.34%. This error decreases with the increase of the frequency. When the frequency varies within the range of 100~1500 Hz (zone-II), the relative error is less than 0.2%. These small errors have demonstrated the close performance agreement between the proposed sensor and the commercial sensor, validating the effectiveness of the presented design.

### 4.4. Characterization of Cross-Sensitivity

**Figure 12:**
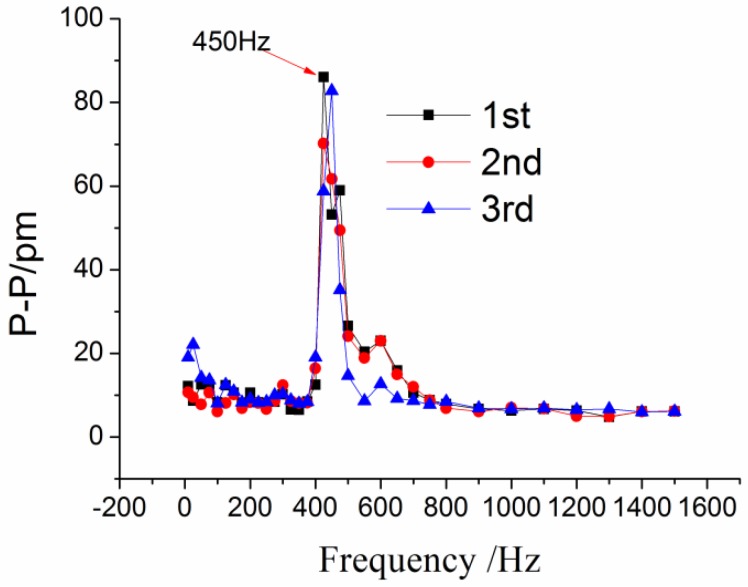
Amplitude-frequency response curves of the #1FBG along the cross direction.

**Figure 13:**
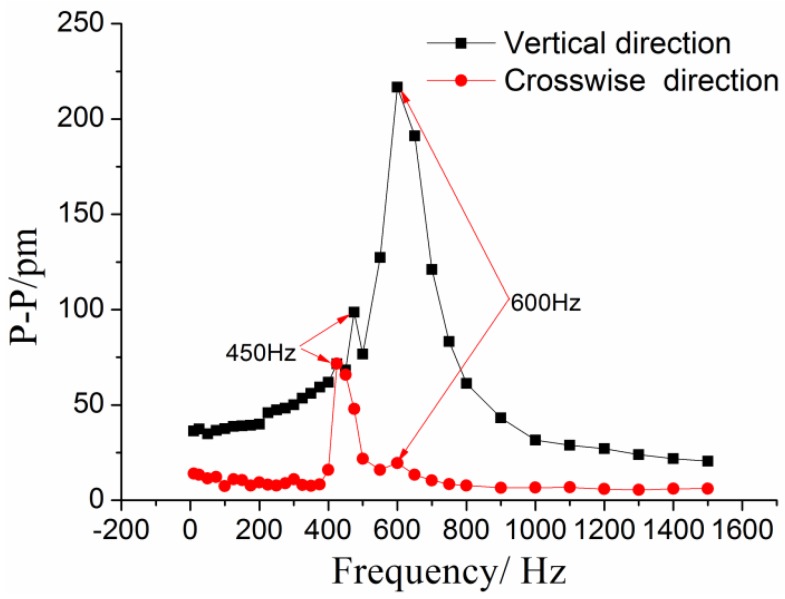
Amplitude-frequency response curves of the cross direction and vertical direction.

Cross-sensitivity is another important indicator to evaluate the vibration sensors. In order to demonstrate the cross-sensitivity for the cross direction, the acceleration amplitude was remained at 10 m/s^2^ with frequency increasing from 10 to 1500 Hz. The frequency-amplitude response curves of the #1FBG along the cross direction are shown in Figure 12. The response curve of the cross-interference is a small constant. Except when the frequency is around 450 Hz. This value is the resonant frequency along the cross direction. The average values from the experimental data were used to calculate the amplitude-frequency response curves along the cross and vertical directions, as shown in Figure 13. The resonant response of the cross direction has been stimulated under the excitation along the main direction. These results suggest that the cross sensitivity is about 3.31% (1/30.1893) compared to the vertical direction, and it is mainly caused by sensor fabrication and assembly errors.

## 5. Conclusions

A novel and practical FBG acceleration sensor that is designed based on the combined configuration of a diaphragm and the axial property of a tightly suspended optical fiber has been proposed and implemented in this work. The proposed decoupling method has also been implemented to realize the separate detection of temperature and acceleration with considering the thermal expansion of the sensor structure. The tightly suspended optical fiber configuration with two fixed ends enables the improvement of the sensor’s sensitivity and repeatability, and avoids the drawbacks associated with the FBG-pasting process. The proposed design has achieved a medium-high working bandwidth with a low cross-sensitivity. Both static and dynamic experiments have been performed to validate the effectiveness of the derived theoretical models and the simulation results, which have effectively supported the parameter determination of the sensor design. The sensor performance can be adjusted by changing the sensor’s mechanical parameters to target different measurements, and its implementation can also be extended to realize multi-point distributed measurements in real-time.

## Figures and Tables

**Table 1:** Characterization of the temperature effects on the two FBGs.

Type	Linearity %	Hysteresis Error/%	Fitted Equation
#1FBG	2.36	8.76	∆*λ*_1_ = 0.00997 × *T* + 1297.63452 (∆*λ*_10_:nm)
#2FBG	1.30	2.04	∆*λ*_2_ = 0.00866 × *T* + 1307.62474 (∆*λ*_2_:nm)

## References

[1] Lee B. (2003). Review of the present status of optical fiber sensors. Opt. Fiber Technol..

[2] Hill K.O., Meltz G. (1997). Fiber Bragg grating technology fundamentals and overview. J. Lightwave Technol..

[3] Majumder M., Gangopadhyay T.K., Chakraborty A.K., Dasgupta K., Bhattacharya D.K. (2008). Fibre Bragg gratings in structural health monitoring—Present status and applications. Sens. Actuators A Phys..

[4] Chen J.J., Liu B., Zhang H. (2011). Review of fiber Bragg grating sensor technology. Front. Optoelectron. China.

[5] Hong C.Y., Zhang Y.F., Zhang M.X., Leung L.M.G., Liu L.Q. (2016). Application of FBG sensors for geotechnical health monitoring, a review of sensor design, implementation methods and packaging techniques. Sens. Actuators A Phys..

[6] Guo Y.X., Zhang D.S., Zhou Z.D., Zhu F.D., Xiong L. (2014). Development and commissioning of FBG sensors for impact test of rock fall protective barrier. Sens. Rev..

[7] Cai L., Tan Y.G., Wei Q. (2015). Nonlinear vibration-FBG sensing technique for plate detection. Sens. Rev..

[8] Wu J., Masek V., Cada M. The possible use of fiber Bragg grating based accelerometers for seismic measurements. Proceedings of the 2nd Canadian Conference on Electrical and Computer Engineering (CCECE’09).

[9] Au H.Y., Khijwania S.K., Tam H.Y. Fiber Bragg grating based accelerometer. Proceedings of the 19th International Conference on Optical Fiber Sensors.

[10] Basumallick N., Biswas P., Dasgupta K., Bandyopadhyay S. (2013). Design optimization of fiber Bragg grating accelerometer for maximum sensitivity. Sens. Actuators A Phys..

[11] Khan M.M., Panwar N., Dhawan R. (2014). Modified cantilever beam shaped FBG based accelerometer with self temperature compensation. Sens. Actuators A Phys..

[12] Liu Q.P., Qiao X.G., Zhao J.L., Jia Z.A., Gao H., Shao M. (2012). Novel fiber Bragg grating accelerometer based on diaphragm. IEEE Sens. J..

[13] Da Costa Antunes P.F., Lima H.F.T., Alberto N.J., Rodrigues H., Pinto P.M.F., de Lemos Pinto J., Nogueira R.N., Varum H., Costa A.G., de Brito Andre P.S. (2009). Optical fiber accelerometer system for structural dynamic monitoring. IEEE Sens. J..

[14] Antunes P., Varum H., André P. (2011). Uniaxial fiber Bragg grating accelerometer system with temperature and cross axis insensitivity. Measurement.

[15] Weng Y.Y., Qiao X.G., Guo T., Hu M., Feng Z., Wang R., Zhang J. (2011). A robust and compact fiber Bragg grating vibration sensor for seismic measurement. IEEE Sens. J..

[16] Li T.L., Tan Y.G., Zhou Z.D. (2016). A Fiber Bragg Grating Sensing-Based Micro-Vibration Sensor and Its Application. Sensors.

[17] Chang Y.T., Yen C.T., Wu Y.S., Cheng H.C. (2013). Using a Fiber Loop and Fiber Bragg Grating as a Fiber Optic Sensor to Simultaneously Measure Temperature and Displacement. Sensors.

[18] Zhang H.R., Lv Q. (2007). Sensor Technical Manual.

[19] Li T.L., Tan Y.G., Wei L., Zheng K., Guo Y.X. (2014). A non-contact fiber Bragg grating vibration sensor. Rev. Sci. Instrum..

[20] Shao R.P. (2005). Mchanical System Dynamics.

